# Application of damage control resuscitation strategies to patients with severe traumatic hemorrhage: review of plasma to packed red blood cell ratios at a single institution

**DOI:** 10.1186/cc13307

**Published:** 2014-03-17

**Authors:** K Jung, K Lee, Y Kim

**Affiliations:** 1 Ajou University School of Medicine, Suwon, South Korea

## Introduction

When treating trauma patients with severe hemorrhage, massive blood transfusions are often needed. Damage control resuscitation strategies can be used for such patients, but an adequate fresh frozen plasma:packed red blood cell (FFP:PRBC) administration ratio must be established.

## Methods

We retrospectively reviewed the medical records of 100 trauma patients treated with massive transfusions from March 2010 to October 2012. We divided the patients into two groups according to the FFP:PRBC ratio: a high-ratio group (≥0.5) and a low-ratio group (<0.5). The patient demographics, fluid and transfusion quantities, laboratory values, complications, and outcomes of both groups were analyzed and compared.

## Results

There were 68 patients in the high-ratio group and 32 in the low-ratio group. There were statistically significant differences between groups in the quantities of FFP, FFP:PRBC, platelets, and crystalloids administered, as well as the initial diastolic blood pressure. When comparing the incidence of complications, bloodstream infections were noted only in the high-ratio group, and the difference was statistically significant (*P *= 0.028). Kaplan-Meier plots revealed that the 24-hour survival rate was significantly higher in the high-ratio group (71.9% vs. 97.1%, *P *< 0.001). The 30-day survival rate was also higher in the high-ratio group (56.2% vs. 67.6%), but the difference was not statistically significant (*P *= 0.117).

## Conclusion

For treating patients with severe hemorrhagic trauma, raising the FFP:PRBC ratio to 0.5 or higher may increase the chances of survival (especially the 24-hour survival rate). Efforts to minimize bloodstream infections during the resuscitation process must be increased.
